# Safety Study of Sodium Pentosan Polysulfate for Adult Patients with Mucopolysaccharidosis Type II

**DOI:** 10.3390/diagnostics9040226

**Published:** 2019-12-17

**Authors:** Kenji Orii, Alaena Lim, Shunji Tomatsu, Molly Stapleton, Yasuyuki Suzuki, Calogera M. Simonaro, Edward H. Schuchman, Toshiyuki Fukao, Tadashi Matsumoto

**Affiliations:** 1 Department of Pediatrics, Graduate School of Medicine, Gifu University, Gifu 501-1193, Japantoshi-gif@umin.net (T.F.); 2 Nemours/Alfred I. duPont Hospital for Children, Wilmington, DE 19803, USA; alalim@udel.edu (A.L.); Molly.Stapleton@nemours.org (M.S.); 3 Medical Education Development Center, Gifu University, Gifu 501-1193, Japan; ysuz@gifu-u.ac.jp; 4 Genetics and Genomic Sciences, Icahn School of Medicine at Mount Sinai, New York, NY 10029, USA; Calogera.Simonaro@mountsinai.org (C.M.S.); edward.schuchman@mssm.edu (E.H.S.); 5 ReqMed Company, Ltd., Tokyo 194-0022, Japan

**Keywords:** PPS, anti-inflammatory factor, mucopolysaccharidosis II, glycosaminoglycan, range of motion

## Abstract

Current therapies for the mucopolysaccharidoses (MPS) do not effectively address skeletal and neurological manifestations. Pentosan polysulfate (PPS) is an alternative treatment strategy that has been shown to improve bone architecture, mobility, and neuroinflammation in MPS animals. The aims of this study were to a) primarily establish the safety of weekly PPS injections in attenuated MPS II, b) assess the efficacy of treatment on MPS pathology, and c) define appropriate clinical endpoints and biomarkers for future clinical trials. Subcutaneous injections were administered to three male Japanese patients for 12 weeks. Enzyme replacement therapy was continued in two of the patients while they received PPS and halted for two months in one patient before starting PPS. During treatment, one patient experienced an elevation of alanine transaminase, and another patient experienced convulsions; however, these incidences were non-cumulative and unrelated to PPS administration, respectively. Overall, the drug was well-tolerated in all patients, and no serious drug-related adverse events were noted. Generally, PPS treatment led to an increase in several parameters of shoulder range of motion and decrease of the inflammatory cytokines, MIF and TNF-α, which are potential clinical endpoints and biomarkers, respectively. Changes in urine and serum glycosaminoglycans were inconclusive. Overall, this study demonstrates the safety of using PPS in adults with MPS II and suggests the efficacy of PPS on MPS pathology with the identification of potential clinical endpoints and biomarkers.

## 1. Introduction

The mucopolysaccharidoses (MPS) are a group of inherited metabolic disorders caused by deficiencies of the individual lysosomal enzyme(s) needed to break down glycosaminoglycans (GAGs). GAGs are long chains of carbohydrates that serve as major components of proteoglycans present in the extracellular matrix (ECM). They function to regulate the movement of molecules through the ECM and to enhance sliding between adjacent tissue; therefore, GAGs are essential to construct all connective tissues, including bones, cartilage, tendons, and skin. However, in the MPS disorders, GAGs accumulate in cells and connective tissues over time, giving rise to progressive damage that affects physical appearance, motor abilities, organ function, and cognitive development [1]. In total, there are 11 known enzyme deficiencies, which give rise to seven distinct MPS types that collectively affect one in every 25,000 births [2,3,4,5,6,7,8]. Depending on MPS type and severity, an affected individual may experience skeletal dysplasia, neurological complications, developmental delay, retinal degeneration, recurrent respiratory infection, and/or cardiac involvement [1].

MPS type II is an X-linked recessive disorder [9] caused by a deficiency of the enzyme iduronate-2-sulfatase, which leads to the accumulation of the GAGs, heparan sulfate (HS) and dermatan sulfate (DS) [10]. Clinical features of MPS II include skeletal abnormalities, such as coarse facial features, joint stiffness, and short stature [11]. Like MPS I and III, MPS II also often includes significant neurological impairment. Patients with a severe form of the disorder have short life expectancy, and often, death occurs in the teenage years due to upper respiratory disease or cardiovascular failure [10].

Several therapies to address the enzyme deficiencies characteristic of MPS have been developed, mainly, enzyme replacement therapy (ERT) and hematopoietic stem cell therapy (HSCT). ERT involves injecting a functional, recombinant form of the defective enzyme(s). It has been successful in alleviating some clinical manifestations of MPS [12] and is approved for MPS I, II, IVA, VI, and VII by the Food and Drug Administration (FDA). However, ERT presents several limitations, such as high cost, need for frequent intravenous infusions, and the potential of eliciting an immune response [13,14,15,16,17]. More importantly, ERT provides limited or no impact on skeletal and neurological manifestations of MPS, most likely due to its limited permeability to avascular cartilage and its inability to cross the blood–brain barrier, respectively [12]. With HSCT, healthy donor cells are transplanted and serve to supply functional enzymes [18,19,20,21,22]. It is the main form of treatment for MPS IH, and can be used for MPS 1H/S, IS, II, IVA, VI, and VII [23,24,25,26,27,28,29,30,31]. However, HSCT entails several complications, such as the limited time to find a matched donor [32,33], the limited number of available donors [34,35], and risk of immune reaction (infection, organ failure graft rejection, and graft-versus-host disease) [26,30,36]. Furthermore, as with ERT, HSCT does not correct some MPS bone manifestations completely [22,32,37,38,39,40]. Therefore, it is important to establish alternative therapies for MPS that are non-immunogenic and can target the brain and bone.

Substrate reduction therapy (SRT) is an alternative approach to treating MPS, and several SRT agents are currently being assessed for therapeutic potential. These include genistein, rhodamine B, and Odiparcil^®^. Instead of addressing the defective enzyme, SRT aims to reduce the biosynthesis of the substrate, in this case, GAGs. Unlike ERT, SRT is theoretically non-immunogenic [12]. Additionally, SRT with small molecules (i.e. genistein)has demonstrated the ability to cross the blood–brain barrier [41,42].

Pentosan polysulfate (PPS) is a semisynthetic, heparin-like glucosaminoglycan of low molecular weight derived from beech trees that has anti-inflammatory and pro-chondrogenic properties. PPS is FDA-approved to treat interstitial cystitis. The mechanism by which treatment occurs in this disorder is not fully established, but PPS is largely thought to replace GAGs at sites of damage in the inner wall of the bladder [43]. PPS can also block histamine release, either by inhibiting the action of mediators that recruit mast cells or by directly inhibiting mast cells themselves [44,45]. Furthermore, PPS is used off-label to treat osteoarthritis because of its pro-chondrogenic properties. PPS also stimulates hyaluronan synthesis by synovial fibroblasts and proteoglycan synthesis by chondrocytes [46,47].

PPS is appealing as an agent to treat MPS since it has positive and extensive human safety data [12]. It also has broad and potent anti-inflammatory properties, and it is well documented that inflammation is an important pathogenic mediator in MPS and other lysosomal storage disorders; it even progresses in patients undergoing ERT [48]. GAG accumulation leads to activation of the toll-like receptor 4 (TLR4) pathway and subsequent release of the inflammatory cytokines, such as tumor necrosis factor-α (TNF-α) and interleukin-1β (IL-1β), which has major pathological effects on the joints and bones [49,50,51].

Based on these observations, PPS treatment led to a lowering of the inflammatory cytokines, TNF-α, interleukin-8 (IL-8), and macrophage inflammatory protein-1α (MIP-1α) in several MPS animal models [52,53,54], as well as IL-1β, TNF-α, and interleukin-6 (IL-6) in in vitro models of Gaucher Disease and Fabry Disease [55]. Treatment with PPS also led to a significant reduction of GAG levels and alleviated MPS pathology in MPS I dogs [54], MPS VI rats [52,53], and MPS IIIA mice [56]. Currently, the mechanism of how PPS leads to GAG reduction in MPS is unknown, but this was observed in multiple animal model studies and in multiple tissues. Furthermore, Hennermann et al. demonstrated the safety of PPS in four adult MPS I patients and showed a reduction in urinary GAGs and some clinical improvements over a six month study period [57]. Each of these patients had received ERT for over one year prior to PPS and was maintained on ERT during the six-month study.

The primary goal of this study was to evaluate the safety of weekly injections in a small cohort of adult Japanese patients with MPS II. This study also aimed to assess the efficacy of PPS treatment on MPS pathology, specifically through the measurement of joint mobility, GAG accumulation, and levels of inflammatory cytokines.

## 2. Materials and Methods

### 2.1. Subjects

Informed consent was obtained for all individuals. The study was approved on (5 August 2014) by the ethics committee on human research at Gifu University (IRB number; 2652–) and followed the ethical principles of the Declaration of Helsinki. Three adult males with attenuated MPS II at 22, 34, and 37 years of age, who will be referred to as Patients 1, 2, and 3 respectively, received weekly injections of PPS for 12 weeks at an initial dosage of 0.5 mg/kg and all subsequent injections at a dosage of 1.0 mg/kg. During the trial, Patients 1 and 2 continued ERT, and for Patient 3, ERT was stopped for two months before the PPS trial. For Patients 2 and 3, injections were administered for 12 consecutive weeks; however, due to an unrelated convulsion experienced by Patient 1 after injection 6, PPS treatment was halted for two weeks and then resumed. Several assessments were performed before, during, and after the clinical trial to assess PPS injections for safety and efficacy. More details about the demographics of each patient are depicted in Table A1 in the Appendix.

### 2.2. Pathophysiological Tests

Blood tests were performed at the start of the clinical trial and continued weekly for 14 weeks (Week 0 to Week 14). Measurements were taken for white blood cell (WBC) count, hemoglobin, platelet count, aspartate aminotransferase (AST), alanine transaminase (ALT), prothrombin time, and activated partial thromboplastin time (APTT). Although the duration of the trial was only 3 months, the following tests were conducted before and after the clinical trial in order to assess if PPS alleviated skeletal symptoms: rating of knee joint pain as reported by the patient on a 100 mm visual analog scale of 0–10 (0 representing “no pain” and 10 representing “the most severe pain imaginable”), 6-min walk test (6MWT), 3-min stair climb test (3MSCT), knee joint cartilage ultrasonography, and shoulder range of motion (ROM) (flexion, extension, adduction, abduction, outer rotation, and inner rotation). Additionally, an electrocardiogram, echocardiogram, ultrasonic cardiography (UCG), brain magnetic resonance imaging (MRI), abdominal computer tomography (CT), auditory brainstem response (ABR) test, pulmonary function test, and visual test were conducted. In general, any adverse digestive, respiratory, cardiovascular, and neurologic symptoms were recorded if detected during the trial.

### 2.3. Assessment of Serum and Urine Glycosaminoglycan (GAG) Levels

Weekly urine and blood serum samples were collected from the start of the clinical trial, before the first injection, to four weeks after the last injection. The urine samples were collected at the same time of day. These blood and urine samples were then analyzed for keratan sulfate (KS), HS, and DS using the liquid chromatography-tandem mass spectrometry (LC-MS/MS) method, as developed by Oguma and Tomatsu in 2007 [58,59,60,61,62,63]. Keratanase II, heparitinase, and chondroitinase B were added to the samples to hydrolyze KS, HS, and DS respectively, into disaccharides. The samples were then purified and analyzed using LC-MS/MS (1290 Infinity Liquid Chromatography with 6460 Triple Quad Mass Spectrometer by Agilent Technologies, Palo Alto, CA) to detect the disaccharides of the individual GAG polymers: monosulfated KS and disulfated KS for KS, DiHS-0S and DiHS-NS for HS, and Di-4S for DS. The concentration of each type of GAG at each time point was compared to the value in untreated subjects without MPS II obtained in the previous study [64].

### 2.4. ELISA for Serum Cytokine Levels

The following serum cytokines were measured before, during, and after the trial via enzyme-linked immunosorbent assay (ELISA): macrophage migration inhibitory factor (MIF), TNF-α, vascular endothelial growth factor (VEGF), IL-1β, IL-8, interleukins 10 and 18 (IL-10, IL-18), monocyte chemotactic protein-1 (MCP-1), and tumor necrosis factor receptor 1 (TNFR1). Measurements were taken at weeks 0, 4, 10, 14, and 17 for Patient 1 and at weeks 0, 4, 8, 12, and 16 for Patients 2 and 3. All measurements were performed in triplicates according to standard protocols using human antibody ELISA kits (R and D Systems, Minneapolis, MN, USA).

## 3. Results

### 3.1. Safety and Adverse Effects

Overall, PPS was well tolerated in the patients, and no serious drug-related adverse events were noted. Patient 1 experienced a convulsion between the sixth and seventh injections. Follow-up electroencephalography (EEG) was conducted and showed mild abnormality with paroxysmal discharge, indicating that the convulsion was likely due to epilepsy. This was the second convulsion that Patient 1 experienced in his life, the first of which occurred approximately five months before the clinical trial began. Thus, it was concluded that the convulsion was not PPS-related. Additionally, Patient 3 experienced a mild elevation of ALT from weeks 6 to 11. At week 6, ALT levels were detected slightly above the normal range (5 IU/L above a range of 5 to 40 IU/L); however, the levels decreased over time and were close to baseline by week 13.

No other adverse effects were detected, except for mild pain and subcutaneous hemorrhage that came with injection. Such effects were temporary and disappeared within two to three days and one to two weeks, respectively. No vomiting, diarrhea, coughing, difficulty of breathing, tachycardia, or chest pain was detected within the duration of the experiment. There were no changes in electrocardiograms for any of the patients. Left atrial to aortic root ratio decreased slightly for Patient 1 and ejection fraction decreased slightly for Patients 1 and 2. According to pulmonary function test results, the overall severity of pulmonary impairment did not change with treatment. Additionally, there were no changes in hearing and visual tests, brain MRI, or abdominal CT scan with treatment. All three patients had retinitis pigmentosa and did not have hepatosplenomegaly before and after the trial. Further details of the tests assessed for adverse effects are depicted in the Appendix, Table A2.

### 3.2. Skeletal Pathophysiology

Although the main objective of this study was to assess the safety of PPS injections in adult MPS II patients over three months, some clinical/pathological parameters were monitored for changes. Patient 1 experienced knee pain and irregularity of knee joint cartilage (as evidenced by ultrasonography) before the initiation of PPS treatment. After the three-month trial, the pain rating decreased slightly from 3 to 1 (on a scale of 10), and cartilage irregularity decreased slightly, as indicated by ultrasound examination. Patients 2 and 3 did not experience any knee pain or cartilage irregularity before the trial, and this did not change with PPS treatment.

Additionally, right and left shoulder flexion increased for Patients 1 and 2, and there was no change for Patient 3. Right and left shoulder extension decreased for Patient 2, and there was no change for Patients 1 and 3. For shoulder abduction, there was an increase observed for the right shoulder of Patient 1 and for both shoulders of Patient 2. There was also an increase in adduction for both shoulders for Patients 1 and 2. The degree of outer rotation decreased for both shoulders in Patient 1 and increased in the left shoulder for Patient 2. The degree of inner rotation increased for the left shoulder of Patient 1 and both shoulders for Patient 2. There was no improvement in ROM for any of these parameters in Patient 3, who was not under ERT during PPS administration. For the 6MWT and 3MSCT, there was a slight decrease in scores for Patients 1 and 2. Measurements could not be obtained for Patient 3 (not applicable, N/A). Measures of these parameters are depicted in Table 1.

### 3.3. Urinary GAG Levels

Overall, large ranges of values were observed for urinary GAGs. No clear trends were observed in all three patients. Additionally, it could not be concluded what kind of change, if any, was induced by PPS treatment, as urinary GAG levels underwent dramatic fluctuations before and after the period of treatment.

Di-4S values had an average range of 246.3% (times baseline) across the three patients. For Patient 1, the change from before PPS treatment to one week after treatment was negative, and for Patients 2 and 3, Di-4S increased with treatment. Across the three patients, DiHS-0S and DiHS-NS values had an average range of 147% and 167.3%, respectively. Similar to Di-4S, HS disaccharides underwent a negative percent change for Patient 1 and a positive percent change for Patients 2 and 3. The average concentration range of monosulfated keratan sulfate (KS) and disulfated KS was 180.3% and 246.3%, respectively. During PPS treatment in Patient 1, disulfated KS underwent a negative percent change, and monosulfated KS underwent a positive percent change. In Patient 2, both monosulfated KS and disulfated KS increased with PPS treatment. In Patient 3, these disaccharides decreased with treatment. For all three patients, urinary GAGs underwent dramatic fluctuations after PPS was discontinued.

For Patients 1 and 2, Di-4S was within mean + 2SD (normal range) at baseline; for Patient 3, Di-4S was above the normal range. These observations did not change with the PPS administration. In Patients 1 and 3, monosulfated KS stayed above and within the normal range, respectively, with treatment. Before, during, and after treatment, DiHS-NS and DiHS-0S was measured to be above the normal range in Patients 1 and 3. For Patient 2, DiHS-0S started within the normal range but was elevated with treatment [64]. The values of disulfated KS varied across all three patients. Results of the urinary GAG levels are summarized in Table A3, Table A4 and Table A5 in the Appendix.

### 3.4. Serum GAG Levels

As with urinary GAGs, large ranges of values were observed for serum GAGs. No clear trends were observed in all three patients. Additionally, it could not be concluded what kind of change, if any, was induced by PPS treatment, as serum GAG levels underwent dramatic fluctuations before and after the period of treatment.

Di-4S values had an average range of 130.3% (times baseline) across the three patients. For Patient 1, the change from before PPS treatment to one week after treatment was positive, and for Patients 2 and 3, Di-4S decreased with treatment. Across the three patients, DiHS-0S and DiHS-NS values had an average range of 91% and 107.7%, respectively. In Patient 2, the percent change from before treatment to one week after the last PPS injection was positive for DiHS-0S and negative for DiHS-NS. HS disaccharides underwent a decrease in Patient 1 and an increase in Patient 3. The average concentration range of monosulfated KS and disulfated KS was 82.3% and 181%, respectively. In Patients 1 and 2, disulfated KS underwent a negative percent change, and monosulfated KS underwent a positive percent change with treatment. Conversely, in Patient 3, monosulfated KS underwent a positive percent change, and disulfated KS underwent a negative percent change. For all three patients, serum GAGs underwent dramatic fluctuations after PPS was discontinued. In general, serum GAGs were initially measured within the normal range, and this was kept during and after treatment. There was, however, an exception observed for monosulfated KS: monosulfated KS was measured above the normal range at baseline, and this did not change with treatment [64]. Results of the serum GAG levels are summarized in Table A6, Table A7 and Table A8 in the Appendix.

### 3.5. Serum Cytokine Levels

**Figure 1:**
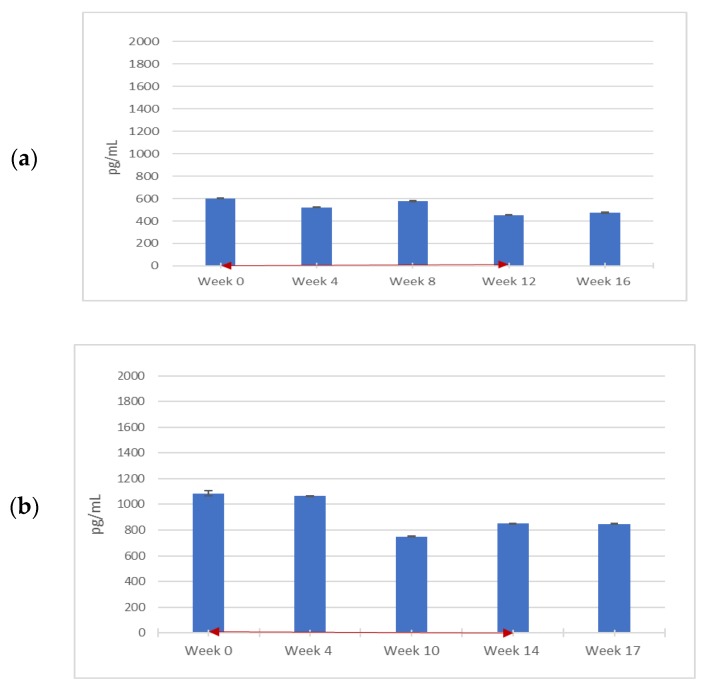

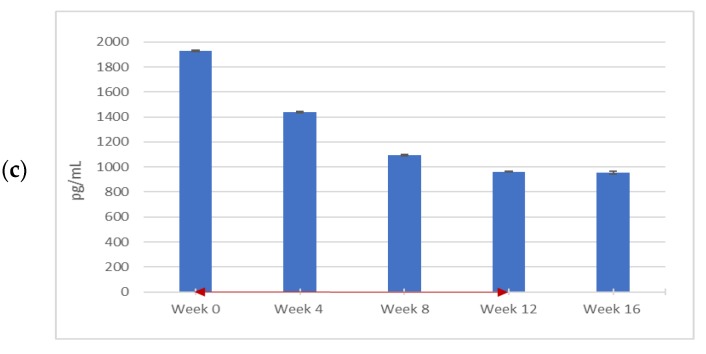
Blood serum migration inhibitory factor (MIF) levels throughout PPS treatment. Three adult males with attenuated MPS II were administered weekly PPS injections for 12 weeks at a dosage of 0.5 mg/kg for the first injection and 1.0 mg/kg for all subsequent injections. Patients 1, 2, and 3, are represented by (**a**), (**b**), and (**c**), respectively. Blood serum samples were measured for MIF levels using a human antibody enzyme-linked immunosorbent assay (ELISA) (R and D Systems) at the start of the clinical trial, before weekly PPS injections, and were then taken every 4 weeks for 16 weeks in Patients 2 and 3 before the injection. For Patient 1, measurements were taken at weeks 4, 10, 14, and 17, as a seizure occurred between injections 7 and 8 and treatment was halted for two weeks. Bars represent standard error of the mean (*n* = 3). The red, double-headed arrows indicate the start and end of the PPS injection period.

Several pro-inflammatory cytokines measured during the trial changed with PPS treatment, these include MIF and TNF-α. Of the two cytokines, only TNF-α levels were elevated above the normal range in the patients before treatment, and there was a trend towards a TNF-α decrease in all three patients, particularly in Patient 2. Of note, even though MIF levels were within or below the normal range at the start of treatment (15.3–52.3 × 10^3^ pg/mL), a trend towards reduction was also noted in the patients with PPS treatment. Again, Patient 2 showed the clearest response. For all other inflammatory cytokines measured, (VEGF, MCP-1, IL-1β, IL-6, IL-8, IL-18, and TNFR1, and anti-inflammatory factor IL-10, no clear changes in the three patients were detected during treatment (). As depicted in Figure 1a−c, the levels generally decreased over time during PPS administration but did not continue to decrease after PPS was discontinued. The decrease in serum levels was most evident in Patient 2.

**Figure 2:**
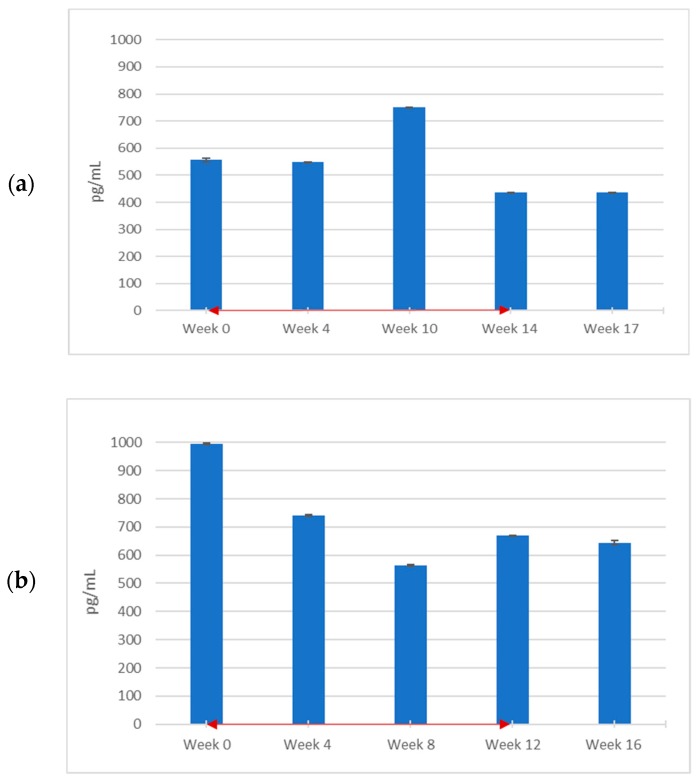

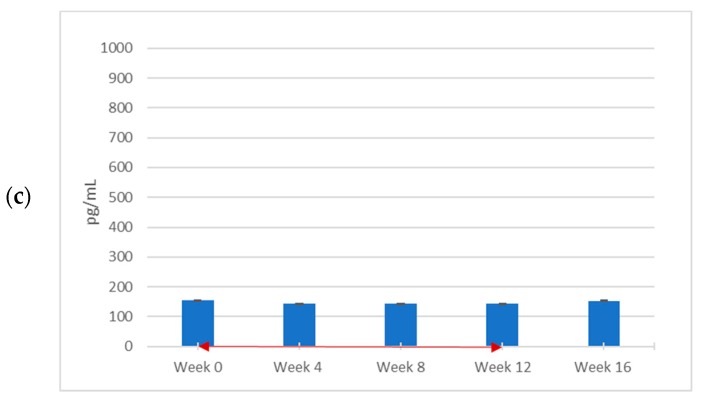
Blood serum tumor necrosis factor-α (TNF-α) levels throughout PPS treatment. PPS injections were administered to 3 adult males with attenuated MPS II weekly for 12 weeks at a dosage of 0.5 mg/kg for the first injection and 1.0 mg/kg for all subsequent injections. Patients 1, 2, and 3, are represented by (**a**), (**b**), and (**c**), respectively. Blood serum samples were measured for TNF-α levels using a human antibody ELISA (R and D Systems) at the start of the clinical trial, before weekly PPS injections and were then taken every 4 weeks for 16 weeks in Patients 2 and 3. For Patient 1, measurements were taken at weeks 4, 10, 14, and 17, as a seizure occurred between injections 7 and 8 and treatment was halted for two weeks. Bars represent standard error of the mean (*n* = 3). The red, double-headed arrows indicate the start and end of the PPS injection period.

With regard to TNF-α, the levels were markedly elevated above the normal range of <15.6 pg/mL at week 0 in all three patients. According to Figure 2a−c, TNF-α underwent a notable decrease over time for Patients 1 and 2 but still remained above normal at the end of the trial period. There was also a transient increase at week 10 in Patient 1. Only a very modest reduction in TNF-α was noted in Patient 3 (who, unlike the other patients, remained off ERT) during PPS treatment, and as with MIF, the most significant result was in Patient 2. After the last injection, levels continued to decrease in Patients 1 and 2. In Patient 3, TNF-α concentration reverted to the baseline value.

## 4. Discussion

The primary aim of this study was to evaluate the safety of weekly subcutaneous PPS administration in adult MPS II patients and to define clinical endpoints and biomarkers for further clinical trials. All patients in the study had attenuated MPS II. As listed in Table A1 in the Appendix, the patients had different iduronate-2-sulfatase mutations. Several mutations (*p*.Arg443Ter, *p*.Asp308Asn, and *p*.Cys171Arg) have been identified previously in attenuated MPS II [65,66,67,68]. *p*.Arg443Ter is a nonsense mutation in a CpG hot-spot of exon 9, and it involves an arginine residue [65]. Both *p*.Asp308Asn and *p*.Cys171Arg are missense mutations, the former is present in exon 7 [65,66,67,68].

Importantly, as in the previous six-month clinical study of attenuated MPS I patients, this study demonstrated an excellent safety profile of PPS in MPS II. As PPS is mainly processed in the liver and can be a weak anti-thrombotic agent [69], potential risks include abnormal coagulation, increased bleeding, and impaired hepatic function. Patient 3 did experience elevation of ALT, a measurement of liver malfunction, during PPS treatment. However, ALT stayed mostly within the normal range and decreased over time; therefore, this effect of PPS was determined to be mild and non-cumulative. There were no signs of abnormal coagulation or increased bleeding. Additionally, Patient 1 experienced convulsions, and injections were halted for two weeks; however, these convulsions were due to epilepsy unrelated to PPS administration. All patients continued treatment for the allotted 12 weeks and none had to withdraw due to adverse effects. No notable digestive, respiratory, cardiovascular, or neurological symptoms occurred with treatment, and this is emphasized by an abdominal CT scan, electrocardiogram, pulmonary function, brain MRI, and hearing and visual assessment results. These findings are significant as they support previous results in MPS I subjects and demonstrate safety in an additional MPS type, MPS II [57].

Given the very short time frame of the study (three months), we did not expect to observe clinical or pathologic improvements in the patients, and patients were not enrolled in the study based on the extent of their clinical or pathologic disabilities. Nonetheless, we did monitor several of these parameters throughout the study. For example, several parameters of skeletal pathology were assessed to determine the efficacy of PPS. Patient 1 experienced decreased knee pain and cartilage irregularity. In Patients 1 and 2, shoulder flexion, abduction, adduction, and inner rotation increased by a range of 6° to 14°, 30° to 68°, 17° to 32°, and 41° to 75°, respectively. However, these changes went unnoticed by the patients themselves, and all other parameters of shoulder ROM did not change or improve. Additionally, there were no improvements in the other tests for mobility (6MWT and 3SCT). Therefore, the findings suggest that PPS had relatively mild efficacy on MPS skeletal pathology during this three-month period. The results also suggest that this efficacy is strengthened when combined with ERT, as Patient 3, who remained off of ERT during PPS administration, experienced less improvement than Patients 1 and 2. It must be cautioned, however, that this was a very short duration-study in only three patients, and more clinical studies are required to assess efficacy.

Although there were mild improvements in skeletal pathology, changes in urine and serum glycosaminoglycans were inconclusive. No common trends were detected across the subjects of the study. Within both the period of treatment and recovery from treatment, patients experienced a great range of GAG levels with respect to baseline. Furthermore, there was often significant variation detected between consecutive time points. These two factors made it difficult to conclude whether a specific GAG (serum or urine) increased or decreased with treatment. This is a notable limitation of the study and is most likely due to the short time frame in which GAG levels were measured and/or individual variability that is particularly emphasized due to the small sample size. As the hallmark of MPS is GAG storage and excretion, and GAG tissue, blood, and urine levels have been used as a biomarker to assess therapeutic efficacy, future studies should utilize a longer treatment and recovery time to allow for the possible action of PPS to take full effect. In addition, a larger sample with wild-type controls should be used to conduct more in-depth statistical analysis when determining the effect of PPS on this parameter of MPS pathology.

Hennermann et al. previously evaluated the effects of PPS injections (at 1 or 2 mg/kg) on four adult patients with MPS I treated for six months in combination with ERT [57]. It was found that there was no significant change in total urinary GAG concentration after 12 weeks of treatment. However, after 24 weeks, total GAG concentrations decreased significantly to near normal values in all four patients [57]. These findings suggest that we may observe a decrease in GAG levels of the MPS II patients if PPS injections are continued for a longer time and/or are administered to patients at higher concentrations. It should also be noted that Hennermann et al. measured total urine GAGs in patients with MPS I [57], while we measured the individual GAG-related disaccharides. As stated before, future studies should assess the efficacy of PPS over a longer period of treatment and recovery time to determine whether administration leads to stabilization and/or a decrease of GAG levels. It is likely that a longer course of treatment is needed to conclusively evaluate clinical changes.

The anti-inflammatory effects of PPS have been extensively documented, and broad anti-inflammatory effects were previously studied in three MPS animal models [52,53,54,56], and also in cells from Fabry and Gaucher disease patients treated with PPS [55]. Several cytokines related to inflammation were also measured during the current trial. MIF and TNF-α levels generally decreased with treatment for all three patients, with Patient 2 responding the most. It should be noted that MIF levels were never elevated above normal range in any of the patients, even at the start of PPS treatment. Also, TNF-α levels, while reduced, did not reach normal levels. The latter observation could be due to the short duration of treatment (three months) and/or the dose of PPS that was administered. It should also be noted that we saw an elevation of cytokine levels when PPS treatment was stopped. Overall, there were decreases observed in TNF-α and MIF with PPS treatment and few changes in any of the other cytokines measured. Additionally, this decrease in TNF-α and MIF was milder in Patient 3, suggesting that the anti-inflammatory activity of PPS in joints might be strengthened when used concurrently with ERT.

The mechanism by which inflammation occurs in MPS has not been fully elucidated, and a hierarchy of relevant cytokines has not yet been fully determined. Out of cytokines studied here, only TNF-α has been implicated in MPS. In the context of the disease, TNF-α contributes greatly to bone pathology [70]. MIF is commonly categorized as pro-inflammatory, like TNF-α [71]; however, unlike TNF-α, it has not been implicated in MPS. Overall, more research with an increased number of patients for a longer period of time is needed to fully establish relationships between PPS treatment, inflammation, and improvement in MPS physiology. It is noteworthy that ROM and pro-inflammatory factors might be useful clinical endpoints and biomarkers respectively, for Phase II/III clinical trials.

Currently, several other agents are being tested for the treatment of MPS, in addition to PPS and ERT. For example, treatment with rhodamine B led to an improvement in behavioral tests for MPS IIIA [72] and MPS I [73] mice, which indicates that rhodamine B has the potential to cross the blood–brain barrier to improve CNS function. It is hypothesized to work as a non-specific inhibitor of GAG synthesis [74]. Not much is known about Odiparcil^®^, but clinical trials are being conducted by Inventiva, testing the agent’s potential as a treatment for MPS VI. Perhaps the most prevalent of the available substrate reduction agents is genistein, which works by regulating expression of GAG synthesis genes [75,76]; genistein has also demonstrated the potential to improve cell cycle defects characteristic of MPS II [77]. Similar to rhodamine B, genistein can cross the blood–brain barrier [41,42].

Among the various experimental therapies being studied in MPS, PPS is somewhat unique since it not only reduces GAG storage but also significantly reduces inflammatory cytokines in MPS animal models. It also appears to cross the blood–brain barrier, although this has only been studied in the context of one neurological MPS model (MPS IIIA) [56]. There is extensive clinical data in different diseases showing the safety of PPS administration in humans, and now seven MPS patients of two different types have been treated (4 MPS I and 3 MPS II) without serious, drug-related adverse events. Thus, we propose that PPS is a very attractive drug that should be studied further in MPS and other lysosomal storage diseases.

In conclusion, this study established the safety and analyzed the potential effectiveness of PPS treatment in three adult male patients with MPS II. There was no serious adverse effect noted throughout PPS treatment, indicating that weekly PPS treatment can be safely used in adults with MPS II. Furthermore, this study has shown that PPS treatment may decrease pro-inflammatory cytokines that are implicated in MPS physiology. Further research should be conducted to fully elucidate the mechanism by which PPS works to decrease inflammation and to confirm its treatment efficacy as a GAG-reducing agent. In the future, testing should be performed over a longer course of time and with a broader range of subjects, including in children, as early intervention is critical in MPS.

## Figures and Tables

**Table 1:** Clinical Outcomes in Mucopolysaccharidoses II (MPS II) Patients before and after Pentosan Polysulfate (PPS) Treatment.

	Patient 1		Patient 2		Patient 3	
	Before	After	Before	After	Before	After
6-Minute Walk Test	495 m	480 m	425 m	410 m	N/A	N/A
3-Minute Stair Climb Test	173 steps	153 steps	188 steps	187 steps	N/A	N/A
Shoulder Range of Motion						
Flexion	R 134, L130	R 140, L 140	R 126, L124	R 132, L138	R 170, L170	R 170, L170
Extension	R 50, L 45	R 50, L 45	R 50, L 45	R 48, L 43	R 30, L 30	R 30, L 30
Abduction	R 90, L 130	R 120, L 130	R 80, L 80	R 128, L 148	N/A	N/A
Adduction	R 28, L 38	R 60, L 55	R 20, L 20	R 52, L 37	N/A	N/A
Outer rotation	R 70, L 90	R 65, L 80	R 5, L 5	R 5, L 6	N/A	N/A
Inner rotation	R 90, L 51	R 90, L 90	R 10, L 10	R 85, L 80	N/A	N/A

## Abbreviations

MPS	Mucopolysaccharidoses
GAG	glycosaminoglycan
ECM	extracellular matrix
HS	heparan sulfate
DS	dermatan sulfate
ERT	enzyme replacement therapy
HSCT	hematopoietic stem cell therapy
FDA	Food and Drug Administration
SRT	substrate reduction therapy
PPS	pentosan polysulfate
TLR4	toll-like receptor 4
TNF-α	tumor necrosis factor-α
IL-1β	interleukin-1β
IL-8	interleukin-8
MIP-1α	macrophage inflammatory protein-1α
IL-6	interleukin-6
WBC	white blood cell
AST	aspartate aminotransferase
ALT	alanine transaminase
APTT	activated partial thromboplastin time
6MWT	6-minute walk test
3MSCT	3-minute stair climb test
ROM	range of motion
UCG	ultrasonic cardiography
MRI	magnetic resonance imaging
CT	computer tomography
ABR	auditory brainstem response
LC-MS/MS	liquid chromotraphy-tandem mass spectrometry
KS	keratan sulfate
ELISA	enzyme-linked immunosorbent assay
MIF	macrophage migration inhibitory factor
VEGF	vascular endothelial growth factor
IL-10	interleukin-10
IL-18	interleukin-18
MCP-1	macrophage migration inhibitory factor
TNFR1	tumor necrosis factor receptor 1
EEG	electroencephalography
N/A	not applicable
VC	vital capacity
TD	tidal volume
FEV1	forced expiratory volume per second
PEF	peak expiratory flow
V.50	maxixmal flow rate of expiration at 50% vital capacity

## Appendix A

**Table A1:** MPS II Patient Demographics.

	Patient 1	Patient 2	Patient 3
Age	22 years old	34 years old	37 years old
Height	135.6 cm	118.2 cm	169.0 cm
Weight	43.9 kg	34.5 kg	58.0 kg
Sex	Male	Male	Male
Iduronate-2-sulfatase Mutations	*p*.Cys1326Thr *p*.Arg443X	*p*.Gly922AlaG922A *p*.Asp308Asn	*p*.Thr511Cys *p*.Cys171Arg
ERT Status	With ERT	With ERT	Without ERT

**Table A2:** Parameters to Assess for Adverse Effects of PPS Treatment in MPS II.

	Patient 1		Patient 2		Patient 3	
	Before	After	Before	After	Before	After
Pulmonary Function Test	Restrictive		Restrictive and obstructive		Normal	
Vital Capacity (VC)	1.66	1.74	1.02		4.09	4.12
Tidal Volume (TV)	0.48	0.56	0.2		0.56	0.77
Forced Expiratory Volume/Second (FEV1)	1.27	1.34	0.63		3.38	3.35
FEV1/VC	79.37%	78.33%	67.74%		83.40%	82.10%
Peak Expiratory Flow (PEF)	2.86	2.99	1.85		Not available	Not available
Maximal Flow Rate of Expiration at 50% VC (V.50)	1.54	1.43	0.66		Not available	Not available
Electrocardiogram	Mild left ventricle hypertrophy	No change	PR prolongation, high amplitude of V5 and V6, T-wave flattening	No change	Normal	Normal
Echocardiogram	Left Atrial to Aortic Root Ratio: 1.31, Ejection Fraction: 66.6%	Left Atrial to Aortic Root Ratio: 1.3, Ejection Fraction 64.5%	Ejection Fraction: 67%	Ejection Fraction: 63%	Not available	Not available
	tricuspid regurgitation, pulmonary regurgitation, mitral regurgitation	No change	left ventricular enlargement, tricuspid regurgitation, aortic regurgitation, mitral regurgitation	No change	aortic valvular hypertrophy, aortic regurgitation	No change
Hearing test	Right:55dB Left:53dB	No change	Right: 73dB Left: 75dB	No change	Not available	Not available
Visual test	Retinitis pigmentosa	No change	Retinitis pigmentosa, Visual field defect	No change	Retinitis pigmentosa, Manual vision	Retinitis pigmentosa, Manual vision
Brain MRI	Bilateral diffuse white matter high signal	No change	Bilateral diffuse white matter high signal	No change	Bilateral diffuse white matter high signal	No change
	Perivascular dilation in subcortical white matter	No change	Perivascular dilation in subcortical white matter	No change	Perivascular dilation in subcortical white matter	No change
	Enlargement of bilateral ventricles	No change	Diffuse patchy high signal in bilateral thalamus, basal nucleus, midbrain, and pons	No change		
Abdominal CT	No hepatosplenomegaly	No change	No hepatosplenomegaly	No change	No hepatosplenomegaly	No change

**Table A3:** Urinary DS levels over the course of PPS treatment with respect to baseline concentration ^a^.

	Patient 1	Patient 2	Patient 3
	Di-4S	Di-4S	Di-4S
Range during Treatment	−55% to 95%	112% to 538%	−99% to 64%
Overall Change with Treatment ^b^	−70%	109%	44%
Changes after Treatment ^c^	Increase to 165% Decrease to −31%	Decrease to −8% Increase to 954%	Increase to 112% Decrease to −23%

^a^ Baseline concentration refers to disaccharide concentration measured before PPS was administered. For example, –70% indicates the value measured at a time point was 70% below baseline. ^b^ Overall change with treatment was determined by calculating the percent change between baseline value and the value measured one week after the last injection. ^c^ Changes after treatment refer to observations made from one week to four weeks after the last injection.

**Table A4:** Urinary HS levels over the course of PPS treatment with respect to baseline concentration ^a^.

	Patient 1		Patient 2		Patient 3	
	DiHS-0S	DiHS-NS	DiHS-0S	DiHS-NS	DiHS-0S	DiHS-NS
Range during Treatment	−32% to 38%	−38% to 35%	44% to 185%	12% to 240%	−100% to 130%	−99% to 102%
Overall Change with Treatment ^b^	−5%	−45%	97%	151%	17%	17%
Changes after Treatment ^c^	Increase to 39% Decrease to −16%	Increase to 30% Decrease to −41%	Decrease to −15% Increase to 225%	Decrease to −18% Increase to 493%	Increase to 94% Decrease to −3%	Increase to 87% Decrease to −29%

^a^ Baseline concentration refers to disaccharide concentration measured before PPS was administered. For example, –5% indicates the value measured at a time point was 5% below baseline. ^b^ Overall change with treatment was determined by calculating the percent change between baseline value and the value measured one week after the last injection. ^c^ Changes after treatment refer to observations made from one week to four weeks after the last injection.

**Table A5:** Urinary KS levels over the course of PPS treatment with respect to baseline concentration ^a^.

	Patient 1		Patient 2		Patient 3	
	Di-KS	Mono-KS	Di-KS	Mono-KS	Di-KS	Mono-KS
Range during Treatment	−70% to 147%	−77% to −37%	25% to 289%	18% to 398%	−94% to 164%	−84% to 37%
Overall Change with Treatment ^b^	82%	−71%	75%	153%	−24%	−36%
Changes after Treatment ^c^	Increase to 175% Decrease to 42%	Increase to 3% Decrease to −61%	Decrease to 42% Increase to 230%	Decrease to 36% Increase to 257%	Increase to 99% Decrease to 18%	Increase to 30% Decrease to 27%

^a^ Baseline concentration refers to disaccharide concentration measured before PPS was administered. For example, 82% indicates the value measured at a time point was 82% below baseline. ^b^ Overall change with treatment was determined by calculating the percent change between baseline value and the value measured one week after the last injection. ^c^ Changes after treatment refer to observations made from one week to four weeks after the last injection.

**Table A6:** Serum DS levels over the course of PPS treatment with respect to baseline concentration ^a^.

	Patient 1	Patient 2	Patient 3
	Di-4S	Di-4S	Di-4S
Range during Treatment	−56% to 54%	−78% to 28%	−45% to 130%
Overall Change with Treatment ^b^	96%	−45%	−47%
Changes after Treatment ^c^	Decrease to −23% Increase to 37% Decrease to −16%	Increase to −18% Decrease to −44% Increase −29%	Increase to 13% Decrease to 3% Increase to 39%

^a^ Baseline concentration refers to disaccharide concentration measured before PPS was administered. For example, 96% indicates the value measured at a time point was 96% above baseline. ^b^ Overall change with treatment was determined by calculating the percent change between baseline value and the value measured one week after the last injection. ^c^ Changes after treatment refer to observations made from one week to four weeks after the last injection.

**Table A7:** Serum HS levels over the course of PPS treatment with respect to baseline concentration ^a^.

	Patient 1		Patient 2		Patient 3	
	DiHS-0S	DiHS-NS	DiHS-0S	DiHS-NS	DiHS-0S	DiHS-NS
Range during Treatment	−53% to 18%	−52% to 76%	−38% to−5%	−44% to 19%	−42% to 127%	−54% to 78%
Overall Change with Treatment ^b^	−16%	−152%	13%	−21%	22%	32%
Changes after Treatment ^c^	Decrease to –24% Increase to 13%	Decrease to 61% Increase to 96%	Decrease to –18% Increase to 33%	Increase to –9% Decrease to –47% Increase to –32%	Increase to 61% Decrease to 21% Increase to 67%	Decrease to 10% Increase to 71%

^a^ Baseline concentration refers to disaccharide concentration measured before PPS was administered. For example, –16% indicates the value measured at a time point was 16% below baseline. ^b^ Overall change with treatment was determined by calculating the percent change between baseline value and the value measured one week after the last injection. ^c^ Changes after treatment refer to observations made from one week to four weeks after the last injection.

**Table A8:** Serum KS levels over the course of PPS treatment with respect to baseline concentration ^a^.

	Patient 1		Patient 2		Patient 3	
	Di-KS	Mono-KS	Di-KS	Mono-KS	Di-KS	Mono-KS
Range during Treatment	−73% to 215%	−45% to 20%	−43%to 2%	−19% to 9%	−43% to 167%	−60% to 94%
Overall Change with Treatment ^b^	−36%	23%	−3%	18%	48%	–51%
Changes after Treatment ^c^	Increase to −24% Decrease to −61%	Increase to 30% Decrease to −16%	Decrease to −28% Increase to −22%	Decrease to −16% Increase to −7%	Increase to 68% Decrease to −19% Increase to −3%	Increase to 3% Decrease to −21% Increase to 42%

^a^ Baseline concentration refers to disaccharide concentration measured before PPS was administered. For example, –5% indicates the value measured at a time point was 5% below baseline. ^b^ Overall change with treatment was determined by calculating the percent change between baseline value and the value measured one week after the last injection. ^c^ Changes after treatment refer to observations made from one week to four weeks after the last injection.
